# Supplementary material for: Rectus femoris electromyography signal clustering: Data-driven management of crouch gait in patients with cerebral palsy
Source: PLoS One. 2024 Aug 20;19(8):e0298945. doi: 10.1371/journal.pone.0298945 (PMC11335125; doi:10.1371/journal.pone.0298945)
Supplement: S1 Appendix — (DOCX) [file pone.0298945.s001.docx]

Supplementary Material (S1 Appendix)

# Hybrid Clustering Algorithm

K-means clustering algorithm starts with randomly initializing ‘K’ centroids in our dataset, and continues by calculating the distance between data point and centroid and assigning each one into the nearest cluster (label), and repeats these until to find the optimal centroids and groups [1]. To solve the main challenge of this algorithm, which is its sensitivity to the initial cluster centroids selection, we developed a hybrid algorithm employing global optimization search approaches, such as particle swarm optimization (PSO). PSO utilizes a swarm (population) of particles to explore promising areas in the search space. Each particle (individual) represents a potential solution and updates itself based on its own experience and that of its neighbors. The algorithm conducts parallel searches using a group of individuals, adjusting their trajectories in a multidimensional space. The motion of particles towards the optimal solution is guided by their position, velocity, previous performance, and the performance of their neighbors [2]. The proposed steps for developing the hybrid PSO- K-means clustering algorithm are as follows [3] [4] :

| ***Algorithm for Hybrid PSO – K-means*** |
| --- |
| 1: Initialize number of clusters as ‘K’ |
| 2: Initialize population size, maximum number of iterations (maxiter) and inertia weight (w) |
| 3: Generate initial population randomly with centroids as its parameters |
| 4: for iter = 0: maxiter, |
| 5: Find fitness function value for all particles |
| 6: Update velocity and position of particles |
| 7: end |
| 8: Apply K-means Algorithm to return the centroids |

To run PSO, initial population size, maxiter and ‘w’, were considered 100, 12000 and 0.72, respectively [5]. The iteration continues till it reaches pre-defined number of iteration or there is no further change in the centroid of the clusters. The hybrid algorithm runs to minimize the sum of the internal Euclidean distance, between the data and the centroids by Equation (1) [6]:

| Eq. 1. | $\mathbf{Objective function=min}\left( \sum_{\mathbf{k=1}}^{\mathbf{K}} \sum_{\mathbf{i=1}}^{\mathbf{n}_{\mathbf{q}}} \mathbf{dist}\left( \mathbf{x}_{\mathbf{i}}\mathbf{-}\mathbf{C}_{\mathbf{k}} \right)^{\mathbf{2}} \right)$ |
| --- | --- |

where $C_{k}$ is the centroid of cluster k, $x_{i}$ is the i-th object of the k-th cluster, K is the number of clusters, and $n_{q}$ is the number of elements on each cluster. $dist$ means Euclidean distance [6].

**References**

1. Rozumalski A, Schwartz MH. Crouch gait patterns defined using k-means cluster analysis are related to underlying clinical pathology. Gait & posture. 2009;30(2):155-60.

2. Van der Merwe D, Engelbrecht AP, editors. Data clustering using particle swarm optimization. The 2003 Congress on Evolutionary Computation, 2003 CEC'03; 2003: IEEE.

3. Rana S, Jasola S, Kumar RJIJoE, Science, Technology. A hybrid sequential approach for data clustering using K-Means and particle swarm optimization algorithm. 2010;2(6).

4. Prakash C, Kumar R, Mittal NJTCJ. Optimized clustering techniques for gait profiling in children with cerebral palsy for rehabilitation. 2018;61(11):1683-94.

5. <https://www.mathworks.com/help/gads/particleswarm.html>.

6. Guerreiro MT, Guerreiro EMA, Barchi TM, Biluca J, Alves TA, de Souza Tadano Y, et al. Anomaly detection in automotive industry using clustering methods—A case study. 2021;11(21):9868.
