# Supplementary material for: Rectus femoris electromyography signal clustering: Data-driven management of crouch gait in patients with cerebral palsy
Source: PLoS One. 2024 Aug 20;19(8):e0298945. doi: 10.1371/journal.pone.0298945 (PMC11335125; doi:10.1371/journal.pone.0298945)
Supplement: Attachment — Submitted filename: Comments.pdf [file pone.0298945.s003.pdf]

Dear Editor,

The manuscript entitled “Rectus Femoris Electromyography Signal Clustering: Data-Driven Management of Crouch Gait in Patients with Cerebral Palsy” has been organized and developed in good shape. The paper investigates the use of electromyography (EMG) cluster analysis on the rectus femoris muscle to enhance gait analysis interpretation in cerebral palsy (CP) patients. By categorizing retrospective gait data into clusters based on EMG signals, the study identifies distinct patterns of muscle activation associated with different gait abnormalities in CP. It suggests that understanding these patterns can improve clinical decision-making and treatment outcomes, highlighting the potential for EMG data to inform rehabilitation strategies and contribute to more personalized care approaches for CP patients. The study is well-developed and the results are intriguing. Once the following comments are addressed, the manuscript is recommended for publication.

1. The manuscript presents a comprehensive analysis of rectus femoris muscle activity during soccer kicking. Could the authors elaborate on the selection criteria for the participants, particularly regarding their skill level and experience in soccer? How might these factors influence the generalizability of the study's findings?

2. The methodology section details the electromyography (EMG) techniques and analysis used. Could the authors discuss any limitations associated with the EMG equipment's sensitivity or the potential for signal interference? How were these factors mitigated to ensure accurate measurements?

3. The Introduction section is clearly developed and easily understandable. However, the coverage of the full lower limb exoskeleton seems somewhat limite. It is advisable to expand upon this topic, providing more comprehensive information about powered full lower limb exoskeletons. In this context, the papers with DOI “10.1109/ACCESS.2023.3325211” and “10.1109/TNSRE.2021.3136088” are strongly recommended as a valuable references.

4. In the results section, significant differences in muscle activation patterns were observed among the various kicking techniques. Could the authors provide insights into the biomechanical implications of these differences for soccer player performance and injury prevention?

5. The discussion highlights the potential applications of this research in sports science and rehabilitation. Could the authors speculate on how these findings could be incorporated into training regimens for soccer players to optimize performance and reduce the risk of injury?

6. The study concludes with suggestions for future research directions, including the analysis of other muscles involved in soccer kicking. Could the authors elaborate on the potential methodologies and technologies that could be employed to expand upon the current study's findings?

7. Considering the dynamic nature of soccer and the variability in kicking techniques, how did the authors account for the consistency of the kicking motion across trials and participants? Could the authors detail any standardization procedures used to minimize variance not attributable to the muscle activation patterns being studied?

8. The manuscript discusses the potential for these findings to influence rehabilitation protocols. Could the authors expand on how the specific muscle activation patterns identified could be translated into targeted exercises or therapies for athletes recovering from lower limb injuries?

9. The study focuses on the rectus femoris; however, soccer kicking is a complex motion involving multiple muscle groups. How do the authors envision the integration of their findings with analyses of other critical muscles (e.g., hamstrings, gluteus maximus) to provide a more holistic understanding of the biomechanics of soccer kicking?
