# Supplementary material for: Rectus femoris electromyography signal clustering: Data-driven management of crouch gait in patients with cerebral palsy
Source: PLoS One. 2024 Aug 20;19(8):e0298945. doi: 10.1371/journal.pone.0298945 (PMC11335125; doi:10.1371/journal.pone.0298945)
Supplement: Attachment — Submitted filename: ResponseToReviewers.docx [file pone.0298945.s004.docx]

PONE-D-24-04264

**Response to reviewers**

**Editor Dr. Narayan**: I just checked and found that one of the reviewers made a typo when mentioning 'soccer kicking' instead of 'gait conditions.' ***The authors are advised to read 'soccer kicking' as 'gait conditions'*** in the comments and try their best to revise the manuscript accordingly. Moreover, they should **indicate about the typo in the 'response letter to reviewers'** and highlight that they have revised the manuscript given different gait conditions (crouch, jump, recurvatum, stiff and mild gait).

**Authors**: We appreciate that the editor took the time to look into this review but we still believe that Reviewer #1 had seen another article which was confused at his side or at the editorial office. This is not just a typo. The answers of reviewers seem cropped. However, we formally address all points below.

**Reviewer #1**: The manuscript entitled “Rectus Femoris Electromyography Signal Clustering: Data-Driven Management of Crouch Gait in Patients with Cerebral Palsy” has been organized and developed in good shape. The paper investigates the use of electromyography (EMG) cluster analysis on the rectus femoris muscle to enhance gait analysis interpretation in cerebral palsy (CP) patients. By categorizing retrospective gait data into clusters based on EMG signals, the study identifies distinct patterns of muscle activation associated with different gait abnormalities in CP. It suggests that understanding these patterns can improve clinical decision-making and treatment outcomes, highlighting the potential for EMG data to inform rehabilitation strategies and contribute to more personalized care approaches for CP patients. The study is well-developed and the results are intriguing.

**Authors**: Thank you for this recommendation. We believe that the reviewer genuinely read our work.

**Reviewer #1**: Once the following comments are addressed, the manuscript is recommended for publication.

**Authors**: Starting here we believe that the review was mixed up potentially be some copy/paste error either at the reviewers’ side or at the editorial office.

1. The manuscript presents a comprehensive analysis of rectus femoris muscle activity **during soccer kicking**. Could the authors elaborate on the selection criteria for the participants, **particularly regarding their skill level and experience in soccer**? How might these factors influence the generalizability of the study's findings?

**Authors**: We cannot address this point as soccer is not being addressed in this publication. There must have been a misunderstanding. (Wrong review?)

2. The methodology section details the electromyography (EMG) techniques and analysis used. Could the authors discuss any limitations associated with the EMG equipment's sensitivity or the potential for signal interference? How were these factors mitigated to ensure accurate measurements?

**Authors**: We are not sure if this is a genuine critique to our work since EMG techniques per se are not addressed in this manuscript. Nevertheless, we modified the following paragraph in discussion:

“Crosstalk remains a major challenge in EMG studies, especially when attempting to isolate the activity of specific muscles. Particularly, the crosstalk from the surrounding vastus lateralis muscle on the activity of RF has recently been extensively reported as a common issue when employing surface EMG, especially in the presence of crouch gait observed in children with CP [39]. However, utilizing wire fine EMG, true activation of RF was noted in 30-45% of crouched gait cycles [39]. Our study specifically focuses on the 20-45% phase of gait in G1 and G2, addressing this unaffected segment of RF activity. Nevertheless, despite lacking access to an extensive database of wire fine EMG data from CP patients, we believe that surface EMG, as a non-invasive and clinically relevant measure of gait in individuals with CP, has the capacity to capture the primary characteristics of muscle activity in these patients.”

3. The Introduction section is clearly developed and easily understandable. However, the coverage of the full lower limb exoskeleton seems somewhat limite. It is advisable to expand upon this topic, providing more comprehensive information about powered full lower limb exoskeletons. In this context, the papers with DOI “10.1109/ACCESS.2023.3325211” and “10.1109/TNSRE.2021.3136088” are strongly recommended as a valuable reference.

**Authors**: Thank you very much for the suggestion. We have added the following paragraph into the introduction:

“EMG can also be utilized to develop improved control strategies for lower-limb exoskeletons, thereby enhancing motor function in patients with CP [10, 11].”

4. In the results section, significant differences in muscle activation patterns were observed among the various kicking techniques. Could the authors provide insights into the biomechanical implications of these differences for soccer player performance and injury prevention?

**Authors**: We cannot address this point as soccer is not being addressed in this publication. There must have been a misunderstanding. (Wrong review?)

5. The discussion highlights the potential applications of this research in sports science and rehabilitation. Could the authors speculate on how these findings could be incorporated into training regimens for soccer players to optimize performance and reduce the risk of injury?

**Authors**: We cannot address this point as sports science is not being addressed in this publication. There must have been a misunderstanding. (Wrong review?)

**Reviewer #2** provides overall satisfaction with the work but emphasizes the need for comparative analysis, discussion of the work's significance and applications, **pictorial representation of the methodology**, and reiteration of the manuscript for coherence and flow.

Overall the work carried is satisfactory. However, few concerns that are needed to be addressed includes:
1. Comparative analysis of existing work with state-of-art research should be included.

**Authors**: Thank you very much for your comment. We have included the following paragraph in the discussion section of our paper:

“Raouafi et al. [40] employed discriminative analysis and K-means clustering techniques to develop a classification approach for determining upper limb disability levels in patients with CP. Their study utilized kinematic and EMG data from a cohort of 13 patients. Similarly, Hu et al. [41] introduced a mixed K-means and hierarchical clustering algorithm to categorize gait patterns in individuals with CP. While methodological studies on gait in CP exist, our study was the first attempt at clustering EMG data in CP using such an extensive clinical dataset. Therefore, to begin, the current research focused only on one muscle (RF) and, further, the crouch condition to investigate the possibility of developing CP-EMG assessment approaches.”

2. The significance of carried work and its application areas are to be discussed in detail.

**Authors**: We appreciate the reviewer's comment. For the first time, our study applied a clustering algorithm to a large database of EMG data related to the rectus femoris muscle in patients with CP and their gait patterns, ultimately leading to the development of a threshold-based treatment decision-making approach for patients exhibiting crouch gait. We have added this paragraph to the discussion section to highlight the novelty and significance of our findings:

“We conclude that clustering EMG data of the rectus femoris has the potential to establish a novel threshold-based treatment decision-making approach for patients with CP, particularly those exhibiting crouch gait.”

3. The more focus of the manuscript should be on addressing the existing challenges.

**Authors**: Thank you very much. In 3D gait analysis in CP, EMG is often measured alongside kinematics and kinetics, yet its utilization for treatment indication remains limited. By employing cluster analysis as a widely used method for identifying trends in datasets, we tried to establish a novel application for EMG in the treatment of gait abnormalities in CP. We think that in the introduction section, particularly in the final paragraph, we have adequately highlighted the existing gap and current challenges in the literature.

4. The authors should elaborate the complete methodology pictorially using flow diagrams, charts etc.

**Authors**: Figure 1 in the manuscript illustrates the procedure utilized in this study through a flow chart. Additionally, the steps proposed to develop the hybrid PSO-K-means clustering algorithm are clearly outlined in the appendix (S1 Appendix.docx).

5. Quality of included figures require consideration.

**Authors**: Thank you very much for the comment. We adhered to the instructions provided on the journal's website to prepare the figures for our submission.

6. Overall manuscript should be re-iterated for complete flow and other issues.

**Authors**: We appreciate the comment and are hopeful that the changes we implemented to address the concerns raised by the reviewer(s) have improved the flow and content of our manuscript.

**Editor:** Funding: German Research Foundation (DFG) (no: WO 1624/ 8-1);

**Authors**: We added the following: “This funder had no role in study design, data collection and analysis, decision to publish, or preparation of the manuscript.”

**Editor**: If there are ethical or legal restrictions on sharing a de-identified data set, please explain them in detail (e.g., data contain potentially identifying or sensitive patient information, data are owned by a third-party organization, etc.) and who has imposed them (e.g., a Research Ethics Committee or Institutional Review Board, etc.). Please also provide contact information for a data access committee, ethics committee, or other institutional body to which data requests may be sent.

**Authors:** We clarified this issue with our ethics committee and got allowance for anonymous publication. We add this section in the manuscript:

“Data Availability:

The data supporting the conclusions of this article will be made available by the authors upon request.”
