# Supplementary material for: Rectus femoris electromyography signal clustering: Data-driven management of crouch gait in patients with cerebral palsy
Source: PLoS One. 2024 Aug 20;19(8):e0298945. doi: 10.1371/journal.pone.0298945 (PMC11335125; doi:10.1371/journal.pone.0298945)
Supplement: Attachment — Submitted filename: ResponseToReviewers-R2.docx [file pone.0298945.s005.docx]

PONE-D-24-04264R1

Rectus Femoris Electromyography Signal Clustering: Data-Driven Management of Crouch Gait in Patients with Cerebral Palsy

PLOS ONE

**Response to reviewers**

6. Review Comments to the Author

Please use the space provided to explain your answers to the questions above. You may also include additional comments for the author, including concerns about dual publication, research ethics, or publication ethics. (Please upload your review as an attachment if it exceeds 20,000 characters)

**Reviewer #1:** The paper has been significantly improved with substantial revisions enhancing both its clarity and depth. The arguments are now more coherently structured, and the evidence is presented in a compelling manner. The methodology has been meticulously refined, and the results are well-supported by the data. Overall, the revisions have added considerable value to the paper, making it a strong candidate for publication. It is recommended that the paper be accepted in its current form.
**Authors:** We appreciate the reviewer's recommendation for the acceptance of our paper.

**Reviewer #3:** This is a very nice study reviewing the utility of attempting to quantify rectus femoris EMG in children with cerebral palsy. I find the use of clustering at specific aspects of gait cycle an interesting concept since the problem of quantitative assessment of EMG has been difficult.

**Some specific comments:**

**Reviewer #3**: 1. In the methods sections there is a note that they used kinematics to define gait patterns however just based on the references it is not clear what these parameters are. Also crouch, jump, and recurvatum are terms typically applied to stance phase while stiff knee is applied to swing phase, and mild gait is none specific, so how do they deal with a patient with mild crouch and stiff knee gait, which is a relatively common problem?
**Authors**: Thank you for the comment. We acknowledge the limitation of our algorithm in considering only the most prominent gait abnormality as the assigned subgroup for each patient. For instance, if a patient exhibits both mild crouch and stiff knee gait, the algorithm would assign the stiff knee gait as the subgroup. We have updated our methods and discussion sections to reflect this limitation accordingly.
Methods:
In addition, considering the hip, knee and ankle angle of the patients in sagittal plane during the gait, they were classified as crouch, jump, recurvatum, stiff knee, and mild gait [14, 24].
Discussion:
Furthermore, the gait subgrouping algorithm we developed, which is based on the hip, knee, and ankle angles in the sagittal plane as outlined in references [14, 24], was limited to identifying patients with one of the following conditions: crouch, recurvatum, jump, stiff, or mild knee gait abnormalities. Although our algorithm was designed to detect the most severe condition as the primary gait issue for a patient, multiple gait abnormalities can occur simultaneously. For future research, we recommend analyzing EMG while considering the severity of each gait abnormality for individual patients.

**Reviewer #3**:  2. In the Methods section there is a clear description EMG signal processing, however on line 142 “Six main features (mean, range, max, min, and their timing) during ten gait phases” is not clear. Does this refer to the mean EMG envelop with in each of the gait segments, or is it defining the kinematic gait segments.
**Authors**: We appreciate the reviewer's comment. These are EMG features we have extracted from the envelopes. we modified the following paragraph in Methods:
Further, six main features (mean, range, max, min, and their timing) from the EMG envelopes were extracted during ten gait phases [6]. These phases included the whole-gait-cycle, stance, swing, loading response, mid-stance, terminal stance, pre-swing, initial swing, mid-swing, and terminal swing phase. These features were used for further analysis.

**Reviewer #3**: 3. There is also a comment in Line 141 “averaged across valid strides” were there a minimum number of cycles required or if only one cycle was available was this used? I also think this should be Steps instead of strides since I presume this is based on right and left cycles separately.
**Authors**: Thank you very much for the comment. We have removed this sentence: “and eventually averaged across valid strides” from the methods and added the following:
The gait events were detected for both right and left sides for at least 7 trials for each individual. The final EMG envelopes were calculated as the averaged time-normalized signals for all the valid strides.

**Reviewer #3**: 4. The lateralis is an important muscle especially in stance phase and is often active when the rectus femoris is not, and in normal gait the two muscles should seldom be active at the same time during normal speed walking ion children with typical development. I would appreciate some comment on the rational for not also evaluating vastus EMG?
**Authors**: Thank you very much for the comment. In order to address your concern regarding the EMG of vastus lateralis we have extended the respective paragraph such that it reads as following: “While methodological studies on gait in CP exist, our study was the first attempt at clustering EMG data in CP using such an extensive clinical dataset. Therefore, the current research focused only on one muscle (RF) and, further, the crouch condition to investigate the possibility of developing CP-EMG assessment approaches. Given that the vastus lateralis is particularly important during the stance phase and is often active when the rectus femoris is not, we suggest including this muscle in combination with the rectus femoris for further EMG analyses in patients with CP.”

**Reviewer #3**: 5. It would be interesting in the discussion to note if and how the authors feel this information could currently be applied to clinical decision making, or if they feel that is still too preliminary.
**Authors**: We appreciate this comment. We acknowledge that this study is our first step in a larger project titled "Muscle Activity in Gait with Cerebral Palsy," funded by the German Research Foundation (DFG) (no: WO 1624/8-1). While we are at the beginning of our understanding of EMG in patients with CP, this study demonstrated that for patients with a crouch knee gait and minimal rectus femoris activity around 85% of the mean EMG in 20-45% of gait cycle (as shown in Table 3 of the manuscript), crouch gait will not be corrected following the intervention. Therefore, EMG can potentially serve as an indicator for treatment outcomes. This finding now motivates our current research question: ‘Does the Electromyogram (EMG) remain unchanged as a fingerprint in patients with cerebral palsy (CP) after orthopedic surgery?’.

We have added the following yellow-highlighted sentence into the conclusion of our manuscript to address your concern on the clinical applicability of our study: “We conclude that clustering EMG data of the rectus femoris has the potential to establish a novel threshold-based treatment decision-making approach for patients with CP, particularly those exhibiting crouch gait. From a clinical point of view, our study demonstrated that, as a rule of thumb for patients with crouch knee gait and minimal rectus femoris activity greater than 85% of the mean EMG during mid-stance of the gait cycle (as shown in Table 3), the crouch gait will not be corrected following the intervention. These findings seem promising and suggest that clustering analyses should be applied on datasets with more muscles. In addition, we mainly evaluated knee movement while other joints and gait abnormalities could also be included in future studies.”
